# Microstructure and Tensile Properties of Graphene-Oxide-Reinforced High-Temperature Titanium-Alloy-Matrix Composites

**DOI:** 10.3390/ma13153358

**Published:** 2020-07-29

**Authors:** Hang Chen, Guangbao Mi, Peijie Li, Xu Huang, Chunxiao Cao

**Affiliations:** 1 National Center of Novel Materials for International Research, Tsinghua University, Beijing 100084, China; chenhang16@mails.tsinghua.edu.cn (H.C.); lipj@mail.tsinghua.edu.cn (P.L.); 2 Aviation Key Laboratory of Science and Technology on Advanced Titanium Alloys, AECC Beijing Institute of Aeronautical Materials, Beijing 100095, China; huangxu621@163.com (X.H.); Chunxiao.Cao@biam.ac.cn (C.C.); 3 Beijing Engineering Research Center of Graphene and Application, Beijing 100095, China

**Keywords:** titanium-matrix composite, graphene oxide, high-temperature titanium alloy, microstructure, tensile properties, silicide

## Abstract

In this study, graphene-oxide (GO)-reinforced Ti–Al–Sn–Zr–Mo–Nb–Si high-temperature titanium-alloy-matrix composites were fabricated by powder metallurgy. The mixed powders with well-dispersed GO sheets were obtained by temperature-controlled solution mixing, in which GO sheets adsorb on the surface of titanium alloy particles. Vacuum deoxygenating was applied to remove the oxygen-containing groups in GO, in order to reduce the introduction of oxygen. The compact composites with refined equiaxed and lamellar α phase structures were prepared by hot isostatic pressing (HIP). The results show that in-situ TiC layers form on the surface of GO and GO promotes the precipitation of hexagonal (TiZr)_6_Si_3_ particles. The composites exhibit significant improvement in strength and microhardness. The room-temperature tensile strength, yield strength and microhardness of the composite added with 0.3 wt% GO are 9%, 15% and 27% higher than the matrix titanium alloy without GO, respectively, and the tensile strength and yield strength at 600 °C are 3% and 21% higher than the matrix alloy. The quantitative analysis indicates that the main strengthening mechanisms are load transfer strengthening, grain refinement and (TiZr)_6_Si_3_ second phase strengthening, which accounted for 48%, 30% and 16% of the improvement of room-temperature yield strength, respectively.

## 1. Introduction

Ti–Al–Sn–Zr–Mo–Nb–Si high-temperature titanium alloys are near-α titanium alloys with excellent high-temperature specific strength and can be used for long-term service at 550–600 °C [1]. Typical examples are IMI834 (Ti–5.8Al–4.0Sn–3.5Zr–0.7Nb–0.5Mo–0.35Si–0.06C, UK), Ti–1100 (Ti–6Al–2.8Sn–4Zr–0.4Mo–0.45Si, US) and TA29 (Ti–5.8Al–4Sn–4Zr–0.7Nb–1.5Ta–0.4Si–0.06C, China) alloy, which have been extensively used in high-pressure compressor blisks and cases of aero-engine [2,3,4]. With the continuous improvement in thrust and thrust–weight ratio of aero-engine, the compressor with higher pressure ratio and working temperature is required. Therefore, there is an urgent demand to enhance mechanical properties of high-temperature titanium alloys [5].

Graphene is a two-dimensional nanomaterial with excellent mechanical properties, including high tensile strength (130 GPa), high Young’s modulus (1.02 TPa) and large surface area (2630 m^2^/g) [6,7,8], which make it suitable as a reinforcement to enhance the mechanical properties of metal materials. Many graphene-reinforced metal-matrix composites were studied. For example, the 2.5 vol% reduced graphene oxide/Cu nanocomposite prepared by Hwang et al. [9] exhibited an excellent yield strength of 284 MPa, which was 80% higher than pure Cu. Yan et al. [10] reported that the yield strength of the 0.5 wt% graphene nanoflakes/Al alloy nanocomposite fabricated by ball-milling (BM) and hot isostatic pressing (HIP) was increased by nearly 50%, while the elongation did not decrease. Zhang et al. [11] found that the tensile strength of Al5083 alloy with only 0.1 wt% graphene increased by 50%. The elongation of FGH96 nickel-based superalloy at 650 °C increased from 18.5% to 26.5% after adding 0.1 wt% graphene [12]. In addition, graphene can also improve the strength and hardness of pure Mg, according to the research of Rashad et al. [13].

Graphene-reinforced metal-matrix composites have shown promising performance in structural applications due to the effective improvement of mechanical properties [14]. However, there are many technical challenges that limit their applications. Dispersion of graphene within the metallic matrices is a key challenge because of the presence of a van der Waals force between the platelets [15]. For graphene-reinforced Ti-matrix composites, BM is the most commonly mixed method to improve the dispersion. For instance, Song et al. [16] prepared the 0.5 wt% graphene/pure Ti composite using BM and spark-plasma sintering (SPS) and its indentation yield strength reached to 918 MPa. Liu et al. [17] reported that the 400 °C compressive yield strength and ultimate compressive strength of 0.25 wt% graphene/pure Ti composite fabricated by BM and SPS increased by 43% and 37%, respectively. Mu et al. [18,19] investigated the mechanical properties of graphene/pure Ti composites produced by BM, SPS and hot rolling. The composite with 0.2 wt% graphene had the highest yield strength of 1010 MPa and tensile strength of 1045 MPa. BM can partly relieve the agglomeration and improve the dispersion of graphene by high impact force and shear force to destroy the van der Waals force between graphene platelets [20,21]. However, the high impact force and shear force simultaneously destroy the structure of graphene and cause the cold welding between Ti particles [17].

Graphene oxide (GO) with the similar two-dimensional nanostructure is an important derivative of graphene, which are decorated with hydroxyl, carboxyl, carbonyl and epoxy groups [22]. The presence of the oxygen-containing groups makes GO sheets strongly hydrophilic and allows to disperse stably in many solvents for a long time [23]. Therefore, it is potential to prepare the mixed powder with GO uniform dispersion by solution mixing method, which can avoid the damage of graphene structure. Dong et al. [24] discovered that GO sheets effectively absorbed on the surfaces of spherical Ti particles by mechanically stirring GO and pure Ti powder in water. Our previous study also suggested that the agglomeration and structural damage of GO can be avoided by mixing GO and titanium alloy powder in anhydrous ethanol [25].

However, graphene-reinforced Ti-matrix composites are mainly focused on pure Ti [16,17,18,19,20,24], Ti6Al4V [26,27,28] and TiAl [29] so far. Very little attention was paid on the influence of graphene or GO on microstructure and mechanical properties (especially tensile properties at high temperature) of high-temperature titanium alloys, which have complex microstructure evolution and high initial strength because of numerous solid solution elements. In this work, high quality GO/high-temperature titanium alloy powders mixed with different addition amounts of GO were prepared by temperature-controlled solution mixing, and then vacuum deoxygenating was subsequently used to decompose the oxygen-containing groups in GO and reduce the introduction of oxygen. Finally, GO-reinforced Ti–Al–Sn–Zr–Mo–Nb–Si high-temperature titanium-alloy-matrix composites were prepared by HIP. The effects of addition amounts of GO on microstructure characteristics and tensile properties were investigated in detail and the strengthening mechanisms were discussed quantitatively.

## 2. Materials and Experiment

### 2.1. Raw Materials

**Figure 1:**
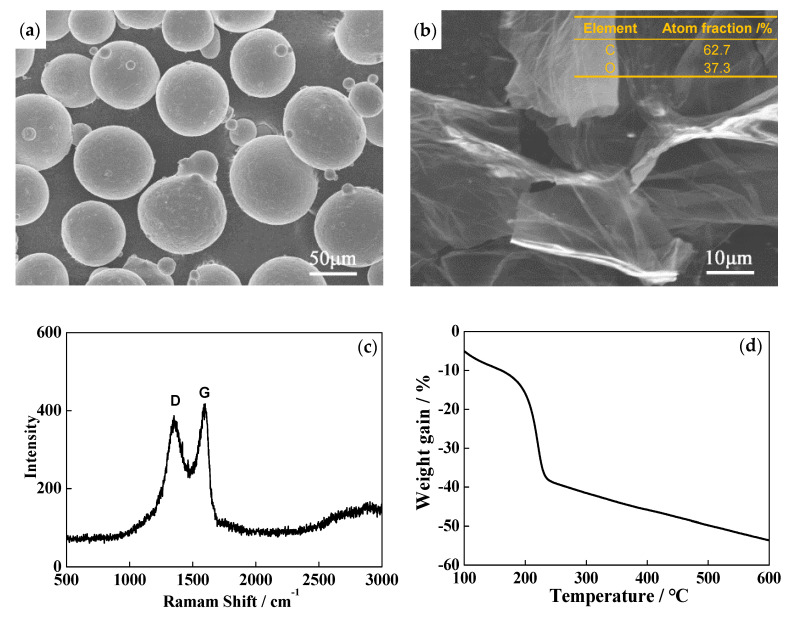
(**a**) SEM image of high-temperature titanium alloy powder; (**b**) SEM image of graphene oxide (GO), the insert table is EDS result of GO; (**c**) Raman spectrum of GO; (**d**) TGA plot of GO.

Ti–Al–Sn–Zr–Mo–Nb–Si high-temperature titanium alloy powder was prepared by AVIMETAL PM Company (Beijing, China) using gas atomization method and the powder with particle diameter of 53–150 μm was screened as the raw material. The chemical compositions of the high-temperature titanium alloy are listed in Table 1. GO was provided by Beijing Engineering Research Center of Graphene and Application (Beijing, China). Figure 1a,b shows the scanning electron microscopy (SEM) images of high-temperature titanium alloy powder and GO. It can be seen that titanium alloy particles are a regular spherical morphology and GO has a large specific surface area and two-dimensional high aspect ratio sheet geometry. From the energy dispersion spectrum (EDS) result in Figure 1b, the C/O atom ratio of GO is ~1.7, indicating a high oxygen content. Raman spectroscopy was applied to characterize the structure of GO, as shown in Figure 1c. GO exhibits a D peak at 1360 cm^−1^ and a G peak at 1597 cm^−1^. The D peak is from the defects in the structure created by the attachment of hydroxyl, carboxyl and epoxy groups on the carbon basal plane; the G peak is corresponding to degree of graphitization. The thermal stability of GO was measured by thermogravimetric analysis (TGA), as shown in Figure 1d. An apparent weight loss is observed between 200–240 °C, which is ascribed to the decomposition of those unstable oxygen-containing groups. This means that O atoms in GO can be removed by thermal treatment.

### 2.2. Fabrication of Composites

**Figure 2:**
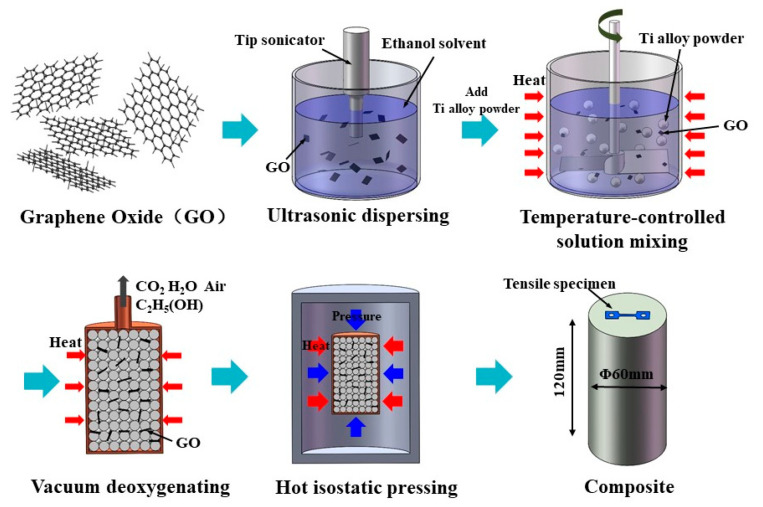
Schematic of GO-reinforced high-temperature titanium-alloy-matrix composite fabrication flow.

The fabrication process of the composites contains four steps: (a) ultrasonic dispersing, (b) temperature-controlled solution mixing, (c) vacuum deoxygenating and (d) HIP, as shown in Figure 2. First, 1 mg/mL GO ethanol solution was sonicated for 30–40 min by tip sonicator to obtain a stable suspension, in which the GO was uniformly dispersed. Then, the high-temperature titanium alloy powder was slowly put into the suspension to form a mixed solution, which was mechanical stirred continuously at 60–70 °C and 300–600 rpm. Stirring was stopped when the mixture was semi-dry because of ethanol volatilization, then dried at 60–80 °C for 30–40 h to obtain GO/high-temperature titanium alloy mixed powders with GO addition amounts of 0, 0.05 wt%, 0.15 wt%, 0.3 wt%, 0.4 wt% and 0.5 wt%. After this, mixed powders were sealed in cylinder-shaped stainless steel cans and evacuated at 400 °C for 10–15 h to deoxygenate. The vacuum condition was always holding at 1 × 10^−4^ pa level. Finally, the canned composite powders were hot-isostatic pressed at 1000 °C and 120–150 MPa for 4 h to obtain GO-reinforced high-temperature titanium-alloy-matrix composites with the size of Φ60 × 120 mm.

### 2.3. Characterization

The morphology and thickness of GO were characterized by SEM (SU-8010, Hitachi, Tokyo, Japan) equipped with an energy dispersive X-ray spectrometer, transmission electron microscopy (TEM, JEM-2100, Jeol, Tokyo, Japan) and atomic force microscopy (AFM, Cypher VRS, Oxford Instruments, Oxford, UK). The chemical structure of GO was measured by Raman spectrometry (JY HR-800, Horiba, Tokyo, Japan). The thermal stability of GO was measured by TGA (TGA/DSC 3^+^, Mettler Toledo, Zurich, Switzerland). The microstructures of composites were observed using optical microscopy (OM, DMI3000, Leica, Solms, Germany), SEM and TEM. The phase compositions of mixed powders and composites were examined by X-ray diffractometer (XRD, SmartLab, Rigaku, Tokyo, Japan). The O and C contents of composites were measured using ONH analyzer (ONH-836, Leco, St. Joseph, MI, USA) and CS analyzer (CS-600, Leco, St. Joseph, MI, USA), respectively. The density were measured by the Archimedes method using electronic balance (YDK03, Sartorius, Göttingen, Germany). The average grain size was measured according to GB/T 6394–2017. Microhardness was measured by Vickers hardness tester (Tukon 2500b, Wilson, Norwood, MA, USA), with the applied load of 500 g (HV 0.5) and a holding time of 15 s. Each sample was measured at least 5 indentations to obtain an average value. The tensile specimens with a gauge length of 10 mm, a width of 2.5 mm and a thickness of 1.5 mm (shown in Figure 2) were cut along the radial direction by wire cut electrical discharge machine (DK77, ZHCF, Suzhou, China) and mechanically ground to the surface roughness of Ra 0.8-μm by surface grinder (M7130, Lingyun, Nantong, China). A microtester (5887, Instron, Norwood, MA, USA) was used to test three tensile specimens in each composition at the room temperature and 600 °C.

## 3. Results

### 3.1. Characterizations of GO and GO/ High-Temperature Titanium Alloy Mixed Powders

**Figure 3:**
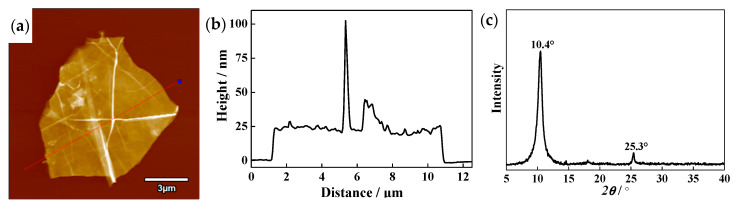
Atomic force microscopy (AFM) image and XRD pattern of GO after ultrasonic dispersing (**a**) top-view image; (**b**) corresponding cross-section height analysis; (**c**) XRD pattern.

Figure 3a shows the AFM image of GO after ultrasonic dispersing. It can be seen that GO sheet is wrinkled and folded due to the small layer thickness, which causes the peaks in Figure 3b. From Figure 3a,b, the width and thickness of GO are ~10 μm and ~25 nm, respectively. Figure 3c presents the XRD pattern of GO after ultrasonic dispersing. A typical characteristic peak of GO is observed at 2θ = 10.4° (interlayer spacing of 8.5 Å). In addition, a low diffraction peak appears at 2θ = 25.3°, which is a shorter interlayer spacing of 3.5 Å, closing to the major peak from (0 0 2) at 2θ = 26.5° corresponding to 3.36 Å of graphite [30]. During ultrasonic dispersing, a few of oxygen-containing groups between interlayers of GO were removed because of the heat produced by ultrasonic waves, resulting in a decrease of interlayer spacing [31].

**Figure 4:**
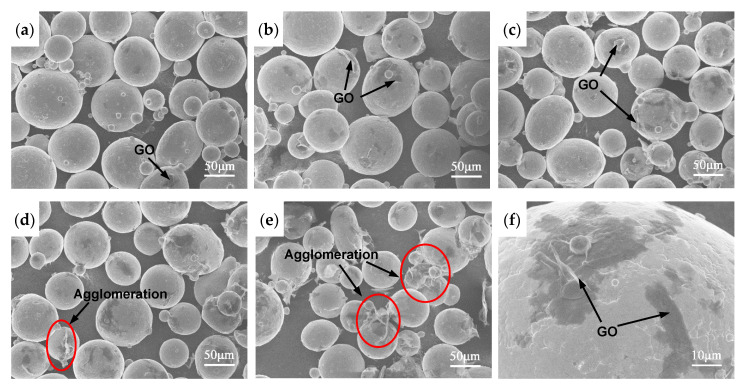
SEM images of the mixed powders with different addition amounts of GO (**a**) 0.05 wt%; (**b**) 0.15 wt%; (**c**) 0.3 wt%; (**d**) 0.4 wt%; (**e**) 0.5 wt%; (**f**) GO adsorbed on the surface of titanium alloy particle.

Figure 4 shows the SEM images of the mixed powders with different addition amounts of GO. It can be seen that the GO disperse uniformly in the matrix powders. The GO thin sheets with original typical wrinkled structure adsorb and wrap on the particle surfaces, as shown in Figure 4f. Few agglomerations are observed when the addition amount of GO is less than 0.3 wt%. It indicates the temperature-controlled solution mixing is a very effective method to prepared high-quality mixed powders when low amount of GO is added.

**Figure 5:**
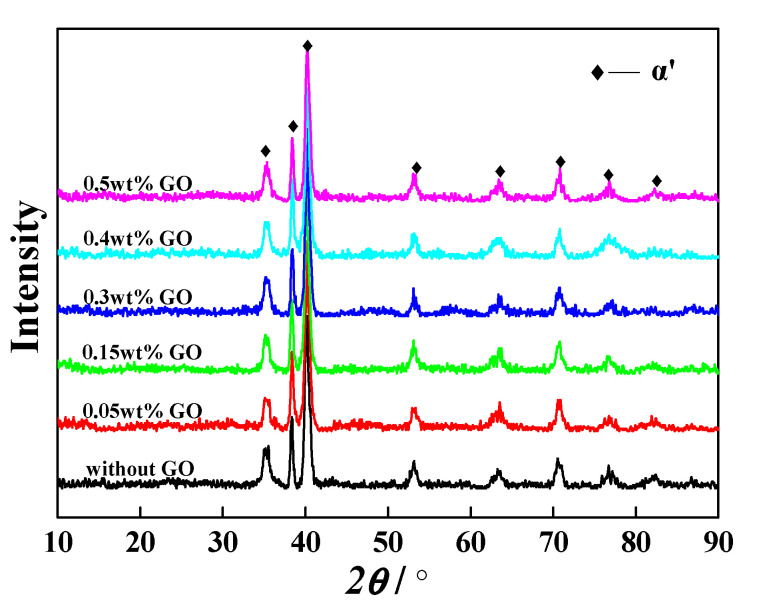
XRD pattern of the mixed powders with different addition amounts of GO.

Figure 5 shows the XRD pattern of GO/ high-temperature titanium alloy mixed powders. The α´ martensite phase of mixed powders is observed. In the gas atomization process, the rapid cooling rate of molten titanium alloy droplets resulted in the formation of metastable α´ martensite phase instead of α phase. No characteristic peak of GO at 2θ = 10.4° or 2θ = 25.3° is detected. It is probably due to the fact that the addition amount of GO is so low (≤0.5 wt%) that XRD cannot identify it.

When GO is heated to above 220 °C, many oxygen-containing groups can be decomposed into CO_2_ and H_2_O [32,33]. Kim et al. [32] reported that most of hydroxyl and carboxyl groups were removed below ~220 °C, and epoxy groups also started to be decomposed at 270 °C. Thus, during vacuum deoxygenating at 400 °C, most of oxygen-containing groups in GO were removed, thus the amount of GO dropped. In order to determine the contents and the C/O atomic ratios of GO in composites after HIP, the contents of C and O elements in composites were measured. The contents of C and O of GO can be considered as the increments of C and O in composite compared with the matrix alloy without GO, and the content of GO is the sum of C and O, the results as shown in Table 2. By calculation, the contents of GO in composites are 46–60% of the original addition amounts when the addition amounts are 0.05 wt%–0.5 wt%. For example, the mass fraction of GO in the composite added with 0.3 wt% GO is 0.17 wt%. The C/O atom ratios of GO in composites are 2.7–3.3, which are much higher than that of raw GO (~1.7). It indicates that a large amount of O element in GO was removed by vacuum deoxygenating, which reduced the introduction of oxygen into composites. In addition, the air and residual ethanol in the mixed powders were removed during vacuum deoxygenating.

### 3.2. Microstructures

**Figure 6:**
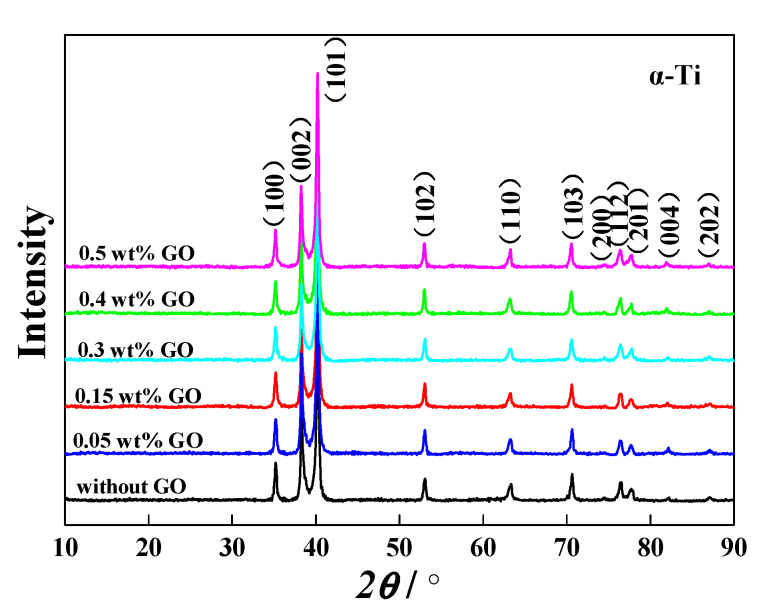
XRD patterns of the composites with different addition amounts of GO.

**Figure 7:**
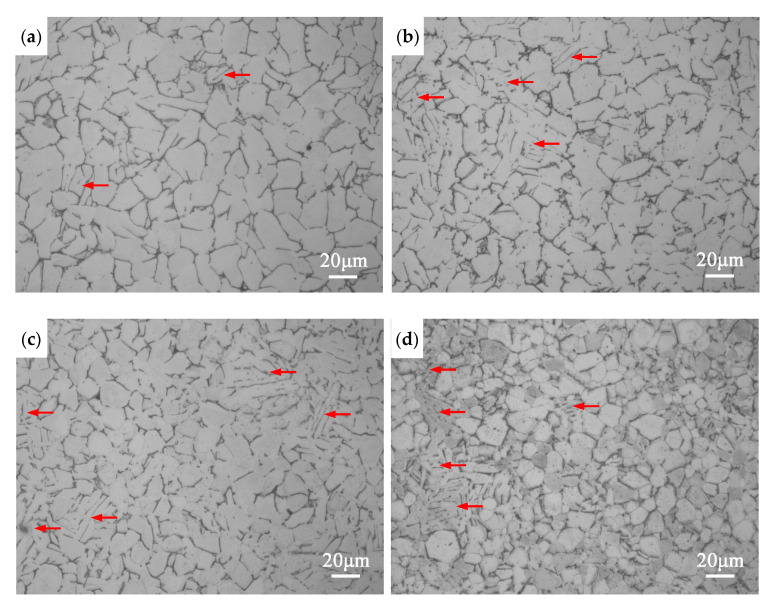
OM images of the composites with different addition amounts of GO (**a**) 0; (**b**) 0.15 wt%; (**c**) 0.3 wt%; (**d**) 0.5 wt%; Lamellar α were marked by red arrows.

The GO-reinforced high-temperature titanium-alloy-matrix composites were fabricated by HIP at 1000 °C. The XRD patterns in Figure 6 shows all the specimens are mainly composed of α-Ti phase. No diffraction peaks of TiC, TiO_2_, graphene or GO are detected, the main reason may be attributed to the small amount of GO. Moreover, the diffraction peaks of β-Ti and silicides are also not observed due to their low compositions. Figure 7 shows the OM images of composites. A large amount of equiaxed α phase and a small amount of lamellar α phase are found in the composites. The content of lamellar α phase increases with the increase of GO addition, even clustered lamellas appear in the composite added with 0.5 wt% GO.

**Figure 8:**
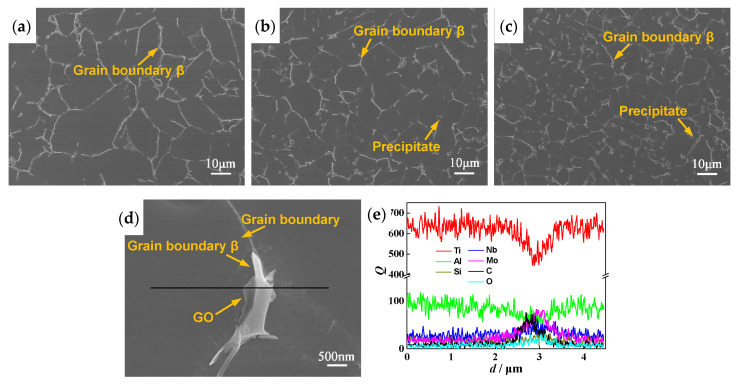
SEM images of the composites with different addition amounts of GO; (**a**) 0; (**b**) 0.3 wt%; (**c**) 0.5 wt%; (**d**) Transparent GO sheet in the composite added with 0.3 wt% GO (**e**) EDS line scanning of the linear area in (**d**).

Figure 8a–c shows the SEM images of composites. The grain size of composite decreases with the increase of GO addition. From Table 2, the average grain size of the composite added with 0.5 wt% GO is 8.5 μm, which decreases by 54% compared with matrix alloy (18.3 μm). There are a small amount of discontinuous β phase located at α grain boundaries and many microscale precipitates dispersed through the composite matrix. The more GO is added, the more precipitates, indicating that GO can promote the precipitation of the second phase particles. The EDS result in Figure 8e shows that the transparent thin sheet located at grain boundary β in Figure 8d has higher C and O contents than matrix, indicating the transparent thin sheet is GO, which has an undamaged sheet structure after HIP. The result of GO located at the grain boundary is consistent with graphene-reinforced pure Ti-matrix composite [18]. From Figure 7 and Figure 8a–c, no porosity is observed, and the previous particle boundaries disappear completely, suggesting that the composites have an excellent compactness after high temperature and high pressure of HIP. As shown in Table 2, the density of composite decreases slightly with GO increases due to the density of GO is less than that of the titanium-alloy-matrix.

**Figure 9:**
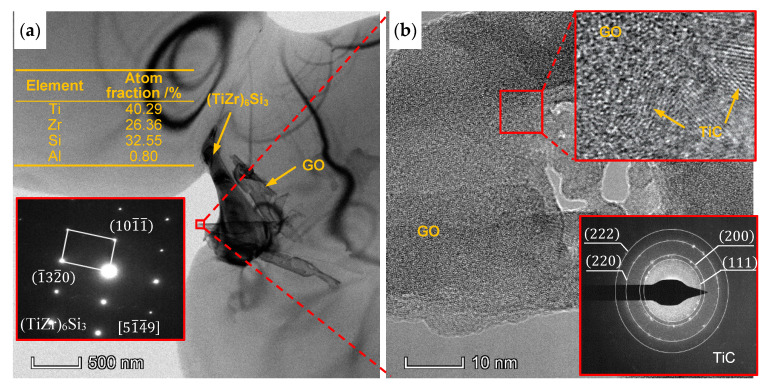
(**a**) TEM image of the composite with 0.3 wt% GO addition, the insert figure and table are the selected area electron diffraction (SED) pattern and EDS analysis result of precipitate; (**b**) HRTEM image of GO; insert figures are the enlarged view and SED pattern of GO.

Figure 9a shows the TEM image of the composite added with 0.3 wt% GO. The GO thin sheet (indicated by yellow dotted line) and the precipitate with the size of ~1 μm are observed. According to the results of selected area electron diffraction (SED) and EDS in Figure 9a, the precipitate is hexagonal S2 silicide (TiZr)_6_Si_3_. The similar S2 silicides were found in near-α titanium alloy prepared by HIP [34]. Figure 9b is HRTEM image of GO, in which some polycrystalline TiC is observed on the surface of GO according to SED pattern. The reaction between Ti and graphene can happen spontaneously in high temperature. The standard free energy ΔG can be expressed as follow [17]:

(1) $$\Delta G=-184,571.8+41.382\;T-5.042T\ln T+2{.425\times 10}^{-3}T^{2}-9{.79\times 10}^{5}/T\;(T<1665.85\;{}^{\circ}C),$$

The ΔG can be calculated to be −174 kJ/mol at 1000 °C which indicates that the spontaneous reaction to generate Ti carbide could occur in HIP step. In this work, unreacted GO with original nanostructure is covered by thin layers of TiC. The TiC reaction layers are tightly bound to the matrix and GO, respectively, which may be helpful to improve the load transfer ability. Mu et al. [19] reported the similar phenomenon of graphene coated with TiC in graphene-reinforced pure titanium-matrix composite fabricated by SPS.

### 3.3. Mechanical Properties

**Figure 10:**
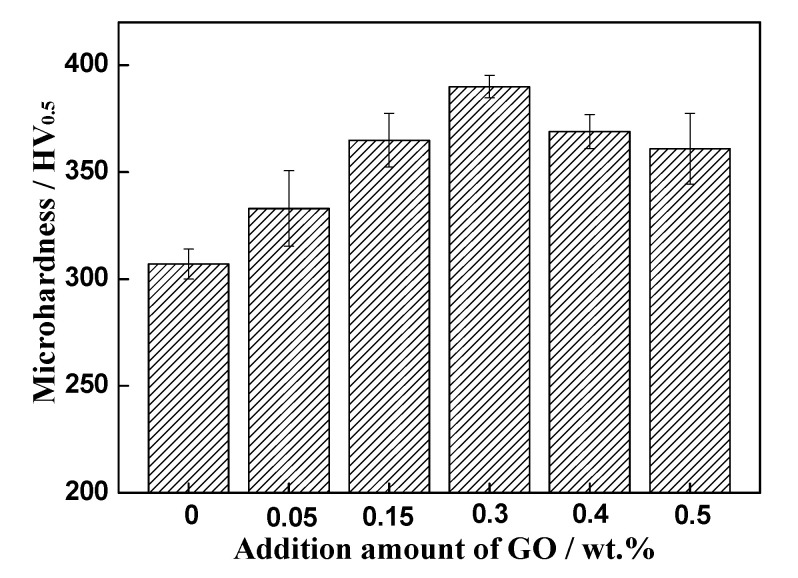
Microhardness of the composites at room temperature.

Figure 10 shows the relationship between room-temperature microhardness and addition amount of GO. The microhardness of the samples increases from 307 HV (without GO) to 390 HV (the composite added with 0.3 wt% GO), which is 27% enhancement. The main reasons for this improvement are as follows: first, the well-dispersed GO thin sheets suppress the grain growth during HIP and restrict the dislocation movement during plastic deformation. On the other hand, a stronger bonding of GO and matrix is formed owing to the formation of TiC flakes (Figure 9b), which helps to increase the microhardness. However, there is a decrease of microhardness when the GO addition is increased over 0.3 wt%. This is because the large agglomerated GO weakens the interface and reduces the density of composite. These results are in good agreements with the reported for GO-reinforced pure Ti-matrix composites [35].

**Figure 11:**
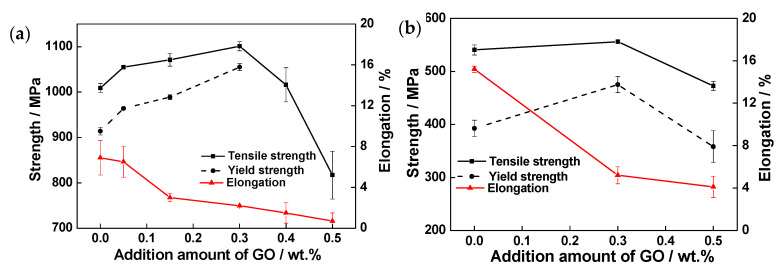
Tensile properties of the composites at different temperatures. (**a**) Room temperature; (**b**) 600 °C.

Figure 11 shows the tensile properties of GO-reinforced high-temperature titanium-alloy-matrix composites at room temperature and 600 °C. Obviously, the strengths at room temperature and 600 °C both increase first, but then decrease. The composite added with 0.3 wt% GO achieves the optimum strengthening effect, the tensile strength and yield strength at room temperature are 1101 MPa and 1055 MPa, respectively, which are 9% and 15% higher than the matrix alloy without GO, and the tensile strength and excellent yield strength at 600 °C are 556 MPa and 475 MPa, respectively, which are 3% and 21% higher than matrix alloy. However, the ductility of composites are declining. The elongations of composite added with 0.3 wt% GO are 2.2% at room temperature and a preferable value of 5.2% at 600 °C. It is noteworthy that the room-temperature yield strengths of the composites added with 0.4 wt% and 0.5 wt% GO are not obtained because of low ductilities.

**Figure 12:**
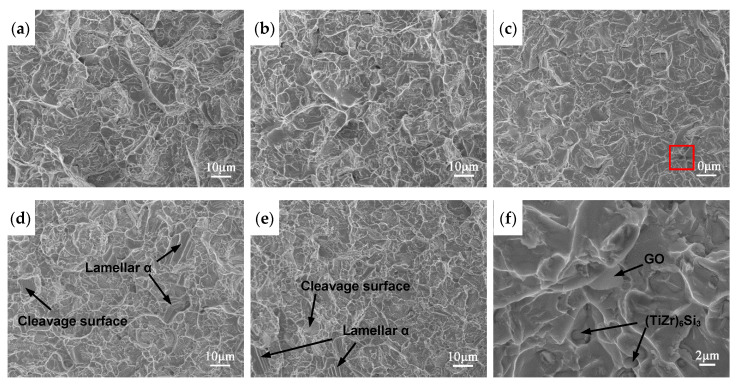
SEM images of tensile fracture surfaces of the composites with different addition amounts of GO at room temperature. (**a**) 0; (**b**) 0.05 wt%; (**c**) 0.15 wt%; (**d**) 0.3 wt%; (**e**) 0.4 wt%; (**f**) high magnification image of rectangular area in (**c**).

Figure 12 shows the fractographs of tensile fractures at room temperature. It can be seen that the fracture surface of the matrix alloy without GO has the largest and deepest dimples and highest tear edges, showing typical ductile fracture characteristics. With the increase of GO addition, the dimples depth and tearing edges height decrease, and a few cleavage surfaces and lamellar grains fracture surfaces appear. Moreover, a large number of (TiZr)_6_Si_3_ hard particles with the size of ~1 μm are found in the fracture dimples of the composites, which can lead to ductility drop. The GO that maintained the original thin sheet structure are pulled out from the matrix (shown in Figure 12f). The stress can be transferred from the matrix to the high strength GO in the process of tensile deformation, thereby improving strength.

## 4. Discussion

The microstructures of GO-reinforced high-temperature titanium-alloy-matrix composites fabricated by solution mixing, vacuum deoxygenating and HIP consist of a large amount of equiaxed α phase, a small amount of lamellar α phase, a few of discontinuous grain boundary β phase, (TiZr)_6_Si_3_ precipitates dispersed uniformly in matrix and GO sheets located at grain boundaries. With the increase of GO addition, the contents of lamellar α phase and (TiZr)_6_Si_3_ precipitates increase and the average grain size decreases significantly.

Recent studies have proved that the equiaxed grains are generated in the high-temperature titanium alloy prepared by HIP due to large plastic deformation and recrystallization [34,36]. For the titanium alloy powder without GO, almost all of the particles have a large plastic deformation to induce the dynamic recrystallization during HIP process, resulting a full equiaxed structure of matrix alloy. However, for GO/ high-temperature titanium alloy mixed powers, the decomposed CO_2_ and H_2_O which are originated from oxygen-containing groups can diffused to the surface of particles to form thin contamination layers because of the high reactivity of titanium. The hard contamination layers hinder the pressure transmission to the inside of the particles [36]. Thus, the microstructure of the inside of the particles forms the lamellar structure due to no sufficient deformation to cause recrystallization. The more GO added, the more lamellar α existed.

Silicides are typical precipitates of high-temperature titanium alloy and the hexagonal S2 silicide (TiZr_0.3_)_6_Si_3_ has been found in the high-temperature titanium alloys prepared by HIP [34] and laser melting deposition [37]. Si dissolves unevenly in the high temperature titanium alloy and tends to segregate and precipitate at dislocations and along α/β phase boundaries [38]. Liu et al. [39] pointed out that dislocations appeared in the titanium matrix near GO due to the different coefficients of thermal expansion (CTE) between matrix and GO or TiC particles. These dislocations can become the nucleation sites of silicides. In addition, the composites with GO addition have more α/β phase boundaries than matrix alloy without GO due to the smaller grains, resulting in more (TiZr)_6_Si_3_ particles.

According to Figure 8a–c and Table 2, the grains of composites are refined markedly after adding GO. Generally, grain size is controlled by nucleation rate and grain growth. The contamination layers in the surface of titanium alloy particles formed in vacuum degassing step were broken sufficiently and could act as nuclei during HIP process, increasing the dynamic recrystallization nucleation rate [40]. On the other hand, GO sheets located at the grain boundaries hindered the atom diffusion and grain boundary migration to inhibit grain growth. Moreover, the pinning effect of (TiZr)_6_Si_3_ hard particles distributed uniformly in matrix could also reduce the grain size.

The strengthening of GO-reinforced high-temperature titanium-alloy-matrix composites is the combined effect of many mechanisms. The possible strengthening mechanisms are grain refinement, load transfer strengthening, the strengthening due to mismatch in CTE and Orowan strengthening.

The relationship between yield strength and grain size can be expressed by Hall–Petch equation [18]:

(2) $$\sigma_{y}{=\sigma }_{0}+kD^{-0.5},$$

where *σ_y_* is the yield strength, *σ*_0_ is the stress that sustain dislocation motion in the interior of a grain, k is constant for a particular material (No study on the constant k of high-temperature titanium alloy is reported, 0.68 MPa∙m^0.5^ of graphene-reinforced pure Ti-matrix composite fabricated by SPS is used here [18]), *D* is the mean grain size. Thus, the improved yield strength of the composites contributed by grain refinement can be obtained from Equation (2) and expressed as:

(3) $$\Delta \sigma_{G}=k({D_{m}}^{-0.5}-{D_{m0}}^{-0.5}),$$

where Δ*σ_G_* is the improved yield strength contributed by grain refinement, *D_m_* and *D_m_*_0_ are the mean grain sizes of the composites with GO addition and the matrix alloy, respectively.

When the composites is subjected to an applied load, stress can be transferred from the matrix to GO through Ti carbide reaction layers by shear stress. The outstanding mechanical properties of GO can support higher loads, thus improving the strength of composites. The shear–lag model is usually used to estimate the strength of titanium-matrix composites [19]. The yield strength (*σ_c_*) can be expressed as:

(4) $$\sigma_{c}{=\sigma }_{r}V_{r}\left(1-\frac{l_{c}}{2l}\right)+\sigma_{m}\left(1-V_{r}\right)\;\left(l\geq l_{c}\right),$$

(5) $$\sigma_{c}{=\sigma }_{r}V_{r}\left(\frac{l}{2l_{c}}\right)+\sigma_{m}\left(1-V_{r}\right)\;\left(l<l_{c}\right),$$

where *σ_m_* is the yield strength of composite matrix, *V_r_* is the volume fraction of GO, *σ_r_* is the fracture strength of GO (24.7 GPa [41]), *l* and *l_c_* are length and critical length of GO, respectively. *σ_m_* can be calculated by:

(6) $$\sigma_{m}=\sigma_{m0}+\Delta \sigma_{G},$$

where *σ*_*m*0_ is the yield strength of matrix alloy, which is 914 MPa at room temperature. The volume fraction of the reinforced phase in composites is always difficult to accurately determine. For this paper, the following equations can be used:

(7) $$\rho_{c}{=V}_{r}\rho_{r}+\left({1-V}_{r}\right)\rho_{m},$$

(8) $$\frac{1}{\rho_{c}}=\frac{M_{r}}{\rho_{r}}+\frac{{1-M}_{r}}{\rho_{m}},$$

where *ρ_c_* and *ρ_r_* are the densities of the composite and GO, respectively; *ρ_m_* is the density of composite matrix, which can be regarded as the density of matrix alloy without GO addition; *M_r_* is the mass fraction of GO. From Equations (7) and (8), *V_r_* can be expressed as:

(9) $$V_{r}=1-\frac{\left({1-M}_{r}\right)\rho_{c}}{\rho_{m}},$$

The critical length (*l_c_*) is the minimum length when GO reaches the fracture strength, which can be calculated as [42]:

(10) $$l_{c}{=\sigma }_{r}\frac{Al}{\tau_{m}S},$$

where *τ_m_* (~0.5 *σ_m_*) is the shear strength of matrix, *A* (= *wt*) and *S* (= 2*(w + t)l*) are cross-section areas of GO and interfacial areas between GO and matrix, *w* and *t* are the width and thickness of GO, respectively. From Figure 3, GO can be simplified to a rectangle of *l* = *w* = 10 μm and *t* = 25 nm for calculation. Calculated by Equation (10), *l_c_* is ~0.67 μm. Thus, *l_c_* < *l*, and Equation (4) is suitable for calculating load transfer strengthening. The improved yield strength (Δ*σ_c_*) contributed by load transfer strengthening can be expressed as:

(11) $$\Delta \sigma_{c}{=\sigma }_{c}{-\sigma }_{m},$$

The CTE of graphene is −8 × 10^−6^ °C^−1^ [43], while the CTE of the high-temperature titanium alloy is 11.6 × 10^−6^ °C^−1^ [44]. There is a significant difference between the CTEs of graphene and the composite matrix. Therefore, the mismatch in CTE can result in prismatic punching of dislocations at the interface, improving the strength of composite [45]. The strengthening of composite due to mismatch in CTE can be expressed as [46]:

(12) $$\Delta \sigma_{\mathrm{CTE}}{=\alpha G}_{m}b\sqrt{\frac{12\Delta T\left(C_{Ti}{-C}_{r}\right)V_{r}}{{bd}_{r}}},$$

where Δ*σ_CTE_* is the improved yield strength due to CTE mismatch, *α* is a constant of 1.25 [46], *G_m_* is the shear modulus of matrix (45.1 GPa [44]), *b* is burger vector of matrix (~0.29 nm [47]), *ΔT* is the change in temperature (980 °C), *C_Ti_* is the CTE of composite matrix (11.6 × 10^−6^ °C^−1^), *C_r_* is the CTE of GO (−8 × 10^−6^ °C^−1^ of graphene is used here), *d_r_* is the average size of GO (10 μm). From this equation, it is clear that the size of GO influences the effect of reinforcement greatly and large GO sheets can weaken the strengthening effected by mismatch in CTE.

When a dislocation encounters a hard second phase particle during motion, it cannot cut, but only bend and bypass the particle, leaving a dislocation loop. Orowan loops around the particles can hinder dislocation motion, resulting in significant work hardening [48]. An increase in yield strength due to Orowan strengthening (Δ*σ_OR_*) is given by [46]:

(13) $$\Delta \sigma_{\mathrm{OR}}=\frac{{0.13G}_{m}b}{d_{r}\left[{\left(\frac{1}{{2V}_{r}}\right)}^{\frac{1}{3}}-1\right]}\ln\frac{d_{r}}{2b},$$

The calculated value of Δ*σ_OR_* is ~0.2 MPa, which is much smaller than the improvement of experimental yield strength. The main reason is that the Orowan strengthening mechanism is suitable for very small hard particles, while the RGO is so large that it is difficult for the dislocations to bypass to form Orowan loops in this work.

**Figure 13:**
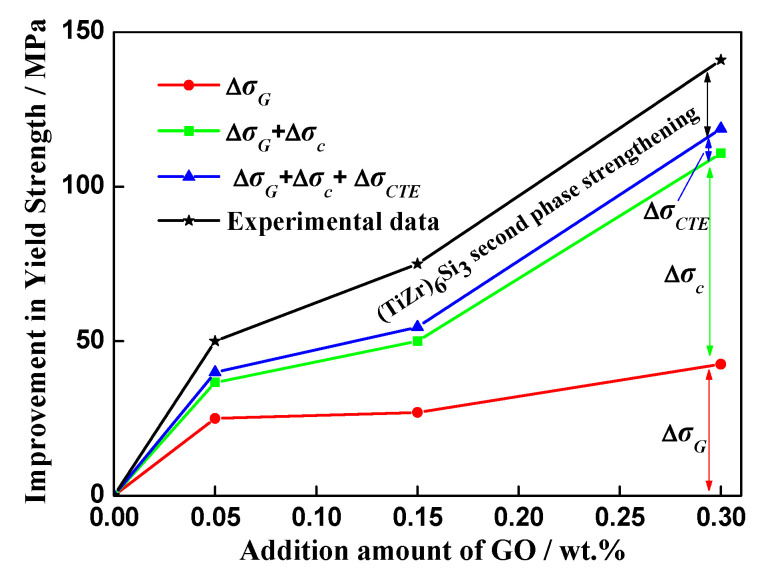
Improvement in yield strength of composites at room temperature.

Figure 13 shows the improvements of experimental dates and theoretical evaluations in yield strength at room temperature. When the addition of GO did not exceed 0.3 wt%, both experimental and theoretical values increase as GO increases. The load transfer strengthening and grain refinement are main strengthening mechanisms in GO-reinforced high-temperature titanium-alloy-matrix composites. The strengthening due to mismatch in CTE and Orowan are weak, which may be ascribed to the large size and low volume fraction of GO. The similar conclusion was reported in graphene/magnesium composite [46]. The value of theory calculation is lower than the experimental date and the residual value can be attributed to the (TiZr)_6_Si_3_ particles. The dislocation entanglement occur when dislocation moved to silicide, which can effectively prevent dislocation slip and improve alloy strength [38]. When the addition amount of GO is 0.3 wt%, Δ*σ_c_*, Δ*σ_G_* and the improvement caused by (TiZr)_6_Si_3_ second phase strengthening are 68.2 MPa, 42.6 MPa and 22.6 MPa, respectively, reaching 48%, 30% and 16% of the total increased value of yield strength.

The decrease in the ductility is a serious problem to limit the application of graphene-reinforced metal-matrix composites, so it is important to find out the reason. For GO-reinforced high-temperature titanium alloy composite, the ductility drop can be attributed to several reasons. It is well known that the microstructure type has a great influence on ductility. Kim et al. [49] pointed out that the ductility of Ti6Al4V alloy with equiaxed phase morphologies was higher than that of the lamellar structure. From Figure 7, it can be found that the percentage of lamellar α grains increases with the increase of GO. In addition, the investigation showed that silicides were detrimental for the ductility [37]. Numerous (TiZr)_6_Si_3_ particles can entangled in dislocations network and cause the drop in ductility. Moreover, the GO sheets with huge specific surface are located at grain boundaries, separate the connection between grains and restrain the deformation of matrix, leading to low ductility [18].

## 5. Conclusions

GO-reinforced Ti–Al–Sn–Zr–Mo–Nb–Si high-temperature titanium-alloy-matrix composites were fabricated by temperature-controlled solution mixing, vacuum deoxygenating at 400 °C and HIP at 1000 °C. The characterizations of mixed powders and the microstructure and mechanical properties of composites were investigated by OM, AFM, SEM, TEM, XRD, tensile test and fracture analysis. Several conclusions can be drawn as follows:

1. The undamaged GO thin sheets adsorb on the surface of titanium alloy particles and disperse relatively uniformly in the mixed powders. Few agglomerations are observed when the addition amount of GO is less than 0.3 wt%. After vacuum deoxygenating, most of oxygen-containing groups in GO are removed and the C/O atom ratio of GO increase from ~1.7 to 2.7–3.3.
2. The microstructure of composites consists of dominant equiaxed α phase, a small amount of lamellar α phase, discontinuous grain boundary β phase, hexagonal (TiZr)_6_Si_3_ precipitates and GO sheets located at grain boundaries. With the increase of GO addition, the contents of lamellar α phase and (TiZr)_6_Si_3_ precipitates increase and grains are refined significantly. The grain size of the composite added with 0.5 wt% GO is 8.5 μm, decreasing by 54% compared with the matrix alloy without GO. The in situ TiC thin layers are produced on the surface of transparent GO sheets, which play a significant role in the good interfacial bonding.
3. When the addition amount of GO is in the range of 0 wt%–0.5 wt%, the composite added with 0.3 wt% GO has the maximum strengths and microhardness. Compared with the matrix titanium alloy without GO, the tensile strength, yield strength and microhardness at room temperature increase by 9%, 15% and 27%, respectively and the tensile strength and excellent yield strength at 600 °C increase by 3% and 21%, respectively. However, the lamellar α-phase and (TiZr)_6_Si_3_ precipitates reduce the ductility of composites.
4. The strengthening mechanisms are attributed to load transfer strengthening, grain refinement and (TiZr)_6_Si_3_ second phase strengthening, which account for 48%, 30% and 16% of the improvement of yield strength at room temperature, respectively. The strengthening due to mismatch in CTE and Orowan are unremarkable.

## Figures and Tables

**Table 1:** Chemical compositions of high-temperature titanium alloy (mass fraction/%).

Al	Sn	Zr	Mo	Nb	Si	C	Ti
5.62	4.12	3.71	0.54	0.71	0.38	0.061	Bal

**Table 2:** Characteristics of GO-reinforced high-temperature titanium-alloy-matrix composites.

Addition Amount of GO (wt%)	Density (g cm^−3^)	Mass Fraction of C (wt%)	Mass Fraction of O (wt%)	Mass Fraction of GO (wt%)	Grain Size (μm)
0 (matrix alloy)	4.596	0.06	0.09	0	18.3 ± 1.4
0.05	4.595	0.08	0.10	0.03	13.7 ± 0.4
0.15	4.594	0.11	0.11	0.07	13.4 ± 0.7
0.30	4.590	0.18	0.14	0.17	11.4 ± 0.8
0.40	4.588	0.20	0.15	0.20	9.6 ± 1.1
0.50	4.585	0.22	0.16	0.23	8.5 ± 0.5
